# Pharmacotherapy and Lung Function Decline in Patients with Chronic Obstructive Pulmonary Disease. A Systematic Review

**DOI:** 10.1164/rccm.202005-1854OC

**Published:** 2021-03-15

**Authors:** Bartolome R. Celli, Julie A. Anderson, Nicholas J. Cowans, Courtney Crim, Benjamin F. Hartley, Fernando J. Martinez, Andrea N. Morris, Holly Quasny, Julie Yates, Jørgen Vestbo, Peter M. A. Calverley

**Affiliations:** ^1^ Pulmonary and Critical Care Division, Brigham and Women’s Hospital, Harvard Medical School, Boston, Massachusetts; ^2^ Research & Development, GlaxoSmithKline, Stockley Park, Middlesex, United Kingdom; ^3^ Statistics & Programming, Veramed Ltd., Twickenham, United Kingdom; ^4^ Research & Development, GlaxoSmithKline, Research Triangle Park, North Carolina; ^5^ Joan and Sanford I. Weill Department of Medicine, Weill Cornell Medicine, New York, New York; ^6^ Division of Infection, Immunity and Respiratory Medicine, The University of Manchester and Manchester University National Health Service Foundation Trust, Manchester Academic Health Science Centre, Manchester, United Kingdom; and; ^7^ Department of Medicine, Clinical Sciences Centre, University of Liverpool, University Hospital Aintree, Liverpool, United Kingdom

**Keywords:** chronic obstructive pulmonary disease, lung function decline, forced expiratory volume, systematic review, spirometry

## Abstract

**Rationale:** Whether pharmacological therapy alters decline in FEV_1_ in chronic obstructive pulmonary disease remains controversial. Because pharmacotherapy improves health status, exacerbation rate, and symptoms, it may be unethical to complete placebo-controlled long-term studies aimed at modifying FEV_1_ decline.

**Objectives:** We conducted a systematic review of placebo-controlled pharmacological trials lasting ≥1 year to address the question of whether therapy alters FEV_1_ decline.

**Methods:** A literature search for randomized trials that included repeated spirometry with at least one active and one placebo arm was conducted. Articles were excluded if study duration was <1 year, <3 spirometric measurements, or <100 subjects per arm. Study design was assessed using the Jadad score. To combine studies and find the estimated effect, we used random effects methodology to account for both within-study and between-study variation.

**Measurements and Main Results:** There were 33,051 patients in the analysis (active component, *n* = 21,941; placebo, *n* = 11,110 in nine studies). The active treatment arms demonstrated a 5.0 ml/yr reduction (95% confidence interval, 0.8–9.1 ml/yr; *P* < 0.001) in the rate of FEV_1_ decline compared with the placebo arms. The relative FEV_1_ differences between active and placebo arms were within the range of differences reported for health status and for the exacerbation rate in the same studies.

**Conclusions:** In chronic obstructive pulmonary disease, pharmacotherapy ameliorates rate of lung function decline. The relative benefit observed is within the range of those reported for health status and exacerbations in the same studies. Guidelines should be adjusted according to these findings.

**At a Glance Commentary**

**Scientific Knowledge on the Subject**

The Global Initiative for Chronic Obstructive Lung Disease (GOLD) states that in patients with chronic obstructive pulmonary disease, pharmacotherapy is effective in reducing symptoms and exacerbations and improving quality of life and exercise endurance but that there is no conclusive evidence that it modifies the long-term decline in lung function. GOLD also states that trials should be specifically designed to test whether pharmacotherapy can achieve such an effect. Given the benefit that medications provide to patients with chronic obstructive pulmonary disease in important outcomes such as exacerbations, dyspnea, and health status, it is unethical to subject symptomatic patients with chronic obstructive pulmonary disease to long-term trials using a placebo control. Owing to these constraints, we completed a systematic review of placebo-controlled pharmacological trials lasting longer than 1 year to answer the question of whether FEV_1_ decline can be ameliorated by pharmacological therapy.

**What This Study Adds to the Field**

This systematic review shows that pharmacotherapy is effective in altering rate of lung function decline. The observed difference of 5 ml per year between active medications and placebo corresponds to a benefit that is similar to the annual differences reported for outcomes (health status and exacerbation rates) that are considered to be improved by the same agents, in the same studies here evaluated. Current guidelines should be adjusted to reflect these findings.

Although the spirometer was first used to determine lung function in 1846, it was not until the late 1950s that the FEV_1_ of an FVC maneuver was proposed as a test to detect the presence of airflow limitation (1). Fletcher and coworkers assessed the change of FEV_1_ over time in men who smoked, linking the rapid decline of that variable to progression of what is now recognized as chronic obstructive pulmonary disease (COPD) (2). The same authors, and subsequent observational and interventional trials, have shown that cessation of smoking results in near normalization of the rate of FEV_1_ decline and thus could change the progression of disease (3, 4). This remains the gold standard to evaluate the evolution of COPD over time.

Pharmacological trials conducted more than 30 years ago using short-acting bronchodilators or inhaled glucocorticoids failed to demonstrate a statistically significantly slower rate of FEV_1_ decline compared with placebo in their intention-to-treat populations (3, 5, 6). Based on these neutral results, the dogma became that the only therapy to alter COPD progression was smoking cessation. Regrettably, this nihilism has negatively affected the public view of this disease and reduced the interest in alternative therapeutic approaches to COPD.

Recent studies provide information that may help explain the lack of effect of pharmacotherapy in these early trials. First, the mean rate of FEV_1_ decline in patients with COPD is lower (4, 7, 8) than that initially reported by Fletcher and colleagues. Second, close to half of subjects with COPD do not have a rate of decline that is steeper than that of healthy smokers and nonsmokers without COPD (9). Therefore, studies evaluating the average change of FEV_1_ of the enrolled subjects have been affected by the inclusion of “normal decliners” that diminish the power to observe an effect of the therapy. Third, the older pharmacological agents used in some of those studies have a short duration of action, and most studies have not been long enough to determine changes in the FEV_1_ for a chronic disease that by definition has a natural history lasting many years.

Fortunately, there have been several randomized trials conducted over recent decades that enrolled a large number of patients, included different long-acting pharmacological agents, have lasted long enough to test the effect of those agents on average FEV_1_, and have included a placebo comparator arm (10–12). Because of the favorable impact of those therapies on exacerbations, health status, and dyspnea, it is unlikely that such studies using a placebo arm will be conducted again as it could be considered unethical to maintain symptomatic patients with COPD on placebo over a long enough time to determine lung function change. In view of this, and prompted by the positive results on FEV_1_ decline in some of the individual trials, we have conducted a systematic review to evaluate the effectiveness of pharmacotherapy on rate of FEV_1_ decline in COPD trials lasting at least 1 year.

## Methods

**Figure 1.**
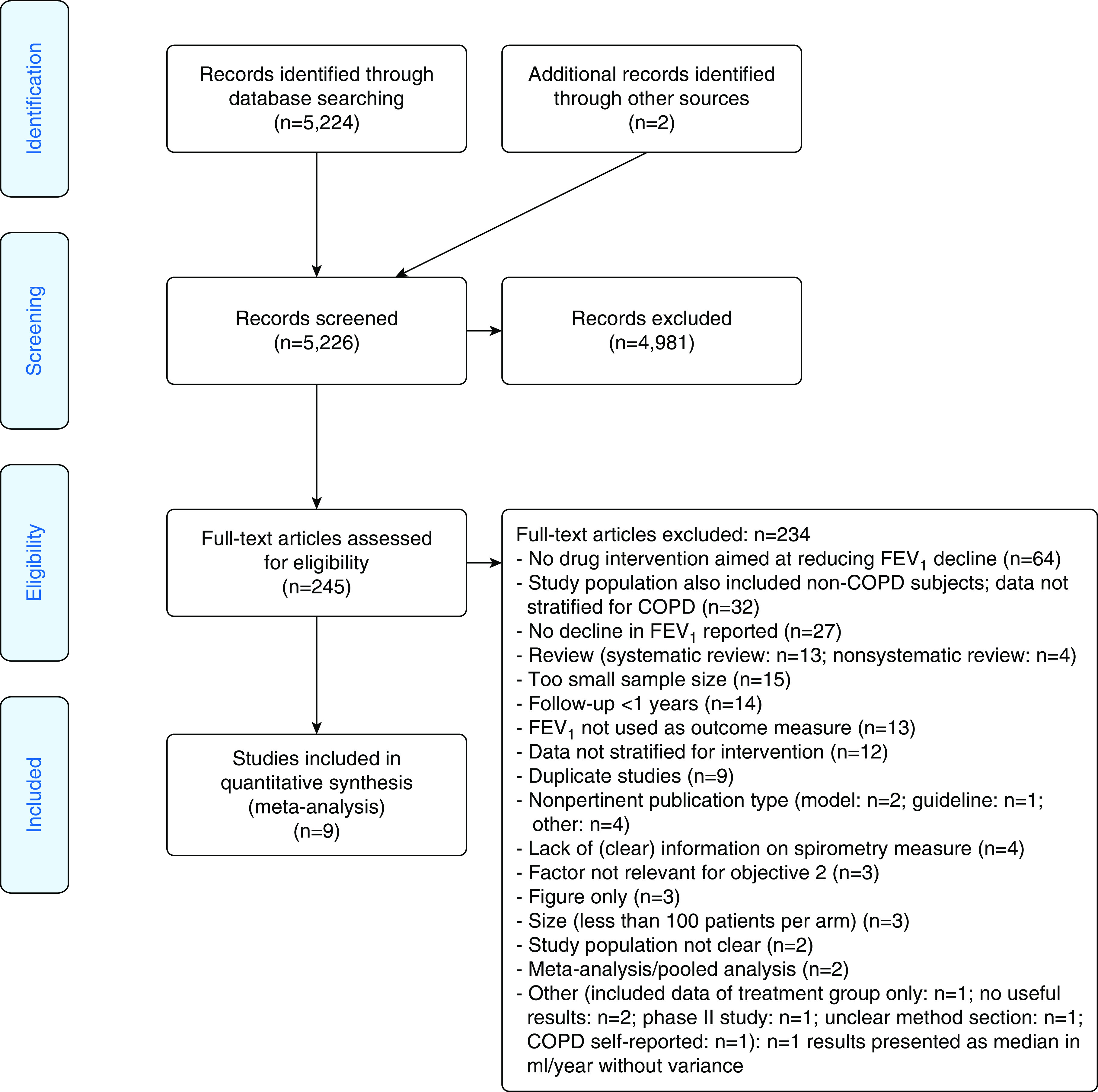
Flow diagram of the systematic review following the Preferred Reporting Items for Systematic Reviews and Meta-Analyses guidelines. COPD = chronic obstructive pulmonary disease.

This systematic review is reported in accordance with the Preferred Reporting Items for Systematic Reviews and Meta-Analyses Statement (13) (Figure 1). A comprehensive literature search for randomized controlled trials (RCTs) lasting at least 1 year and enrolling at least 100 patients per arm that included measurement of spirometry as one of the outcomes and at least one arm of an active medication and one with placebo were reviewed. Three authors (B.R.C., J.Y., and H.Q.) independently checked the relevant RCTs found from the literature. RCTs were selected in agreement with the previously mentioned criteria, and any difference in opinion about eligibility was resolved by consensus.

### Study Selection

A systematic review of the literature was conducted to identify clinical trials designed to assess the effect of an intervention on decline in FEV_1_ in patients with COPD (Figure 1). Initially, a PubMed search conducted through 2017 identified relevant articles based on the following search criteria: COPD diagnosis, decline in lung function/FEV_1_, and study design (Table E1 in the online supplement). Additional records published after the initial search date until December 2019 were identified. The resulting articles underwent a three-step review to identify the studies relevant for this analysis. There was an initial screening of each title and abstract to confirm whether data were relevant for this research objective. The full text of articles selected in the initial review were assessed and excluded if they met any of the following criteria: nonpertinent publication type, duration of study less than 1 year, fewer than three spirometry assessments, fewer than 100 subjects per arm, non–physician-diagnosed COPD, lack of quantitative data, or failure to present FEV_1_ decline in ml/yr as well as the variance for the FEV_1_. Additional exclusions occurred during the data extraction phase if applicable information was not available.

### Outcomes

The main outcome of interest was the difference in rate of FEV_1_ decline in ml/yr between the active pharmacological therapy compared with placebo. In a secondary analysis, we explored differences between individual medication components and placebo on the same outcome. We also present the differences in exacerbation rates and St. George’s Respiratory Questionnaire (SGRQ) scores between the active and the placebo arms in the same studies, to help interpret the relative benefit of the absolute treatment difference observed in FEV_1._

### Quality Score and Risk of Bias Assessment

The Jadad score, with a scale of 1 to 5 (with 5 being highest), was used to assess the quality of the papers concerning the likelihood of bias related with randomization, double blinding, withdrawals, and dropouts (14).

### Statistical Analysis

To combine the studies to find the overall estimated effect, we used random effects methodology to account for both within-study and between-study variation (15). The therapies were compared and the treatment differences and 95% confidence intervals (CIs) were presented in a Forest plot. We performed four separate *post hoc* analyses to investigate the effect of the different classes of therapies on the rate of decline in FEV_1_ (ml/yr). These were active versus nonactive, excluding the BRONCUS (Bronchitis Randomized on NAC Cost-Utility Study) study (16), which used the oral agent *N*-acetylcysteine as the active medication; inhaled corticosteroid (ICS) versus non-ICS; and long-acting bronchodilators (LABDs), either long-acting muscarinic antagonist or long-acting beta adrenergic bronchodilators, versus arms without bronchodilators (non-LABD); in the UPLIFT (Understanding Potential Long-Term Impacts on Function with Tiotropium) study, 60% of patients in both arms were receiving long-acting β-adrenergic agents. No adjustments were made for multiplicity. To investigate the effect of ICS versus non-ICS, in studies with more than one ICS arm, these ICS arms were combined (e.g., in SUMMIT [Study to Understand Mortality and Morbidity], we pooled fluticasone furoate [FF] and FF/vilanterol to be in the ICS arm). Means and SEs were extracted from the publications, and where necessary, 95% CIs were used to derive SEs. We calculated the Q statistic and from this the *I*^2^ statistics to quantify the heterogeneity. SAS version 9.4 (SAS Institute) was used for all analyses.

## Results

### Study Characteristics

A total of 33,051 patients with COPD were included in the analysis (active component, *n* = 21,941; placebo, *n* = 11,110) selected from nine published studies (3, 6, 11, 16–21). One study used tiotropium, one used fluticasone furoate/vilanterol (FF/vilanterol) and the components, one fluticasone propionate/salmeterol and the components, one used fluticasone propionate alone, one used budesonide, one used triamcinolone, one used *N*-acetylcysteine, and one used ipratropium bromide. All studies were published between 1994 and 2018, and relevant patient demographics and study characteristics are summarized in Table 1. The mean Jadad score for the nine studies was 4.8. All trials were randomized and double-blind and with period of treatment and observation that ranged from 40 months to 5 years.

**Table 1.** Baseline Demographics and Characteristics of the Patients Included in the Systematic Review of Pharmacological Trials Lasting 12 Months or Longer in Patients with COPD

	(1997–2003) BRONCUS* Ref 16	(1992–1998) ISOLDE* Ref 6	(2000–2005) TORCH* Refs 10 and 18	(2003–2008) UPLIFT* Ref 11	(1986–1994) Lung Health I* Ref 3	(2011–2015) Zhou et al.* Ref 19	(2011–2015) SUMMIT* Refs 12 and 20	(1992–1994) Copenhagen City Lung Study* Ref 17	(1994–1999) Lung Health Triamcinolone* Ref 21
Treatment arms: placebo/**active intervention(s)**	Placebo/***N*-acetylcysteine**	Placebo/**fluticasone propionate**	Placebo/**salmeterol/fluticasone propionate/salmeterol + fluticasone propionate**	Placebo/**tiotropium**	Placebo/**ipratropium bromide**	Placebo/**tiotropium**	Placebo/**fluticasone furoate/vilanterol/fluticasone furoate + vilanterol**	Placebo/**budesonide**	Placebo/**triamcinolone acetonide**
Number of participants in study (*and in RoD analysis, if different*), (total) placebo/**total active intervention**	(523) 267/**256**	(751) 375/**376***InRoD analysis*: (664) 325/**339**	(6,184) 1,545/**4,639***InRoD analysis*: (5,343) 1,261/**4,082**	(5,992) 3,006/**2,986***InRoD analysis*: (4,964) 2,410/**2,554**	(3,923) 1,962/**1,961**	(771) 383/**388**	(16,485) 4,111/**12,374***InRoD analysis*: (15,457) 3,800/**11,657**	(290) 145/**145**	(1,116) 557/**559**
Age, mean, yr	62	64	65	65	48	64	65	59	56
Sex, F, *n* (%)	110 (21)	191 (25)	1,263 (24)	1,520 (25)	2,186 (37)	113 (15)	4,196 (25)	115 (40)	412 (37)
Smoking status current, %	46%	38%	44%	30%	NA	41%	47%	77%	90%
Previous year exacerbation hx, yearly rate	Placebo: 2.5Active: 2.4	NA	0: 43%	NA	NA	NA	0: 61%	NA	NA
			1: 25%				1: 24%		
			≥2: 32%				≥2: 15%		
FEV_1_, mean, L^†^	1.65	1.41	1.24	1.3	2.75	1.9	1.7	2.4	2.3
FEV_1_, mean, % predicted	57	50	45	48	75	78	60	87	68
Study duration	3 yr	3 yr	3 yr	4 yr	5 yr	2 yr	1.8 yr^‡^	3 yr	40 mo^§^
Number of spirometric assessments	13	12	5	17	5	6	7^‖^	12	Every 6 mo^¶^
Completion of the study placebo/**total active intervention**	168/**186**	175/**212**	55%/**63%**	1,648/**1,887**	>94%**	282/**303**	2,919/**9,311**	94/**109**	92%

*Definition of abbreviations*: BRONCUS = Bronchitis Randomized on NAC Cost-Utility Study; COPD = chronic obstructive pulmonary disease; hx = history; ISOLDE = Inhaled Steroids in Obstructive Lung Disease in Europe; NA = not available; Ref = reference; RoD = rate of decline in FEV_1_; SUMMIT = Study to Understand Mortality and Morbidity; TORCH = Toward a Revolution in COPD Health; UPLIFT = Understanding Potential Long-Term Impacts on Function with Tiotropium.

Statements and numbers in bold reflect the active arms of the study.

* Date is start of recruitment to study complete.

^†^ Post-bronchodilator FEV_1_ except for Lung Health I and Copenhagen City Lung Study.

^‡^ Study was event-driven average treatment in years.

^§^ Variable follow-up.

^‖^ 15,457 contributed an average of seven post-bronchodilator assessments at 3-month intervals.

^¶^ Every 6 months during follow-up.

** Completed fifth year of spirometry.

### Impact of Pharmacotherapy on FEV_1_ Decline

**Figure 2.**
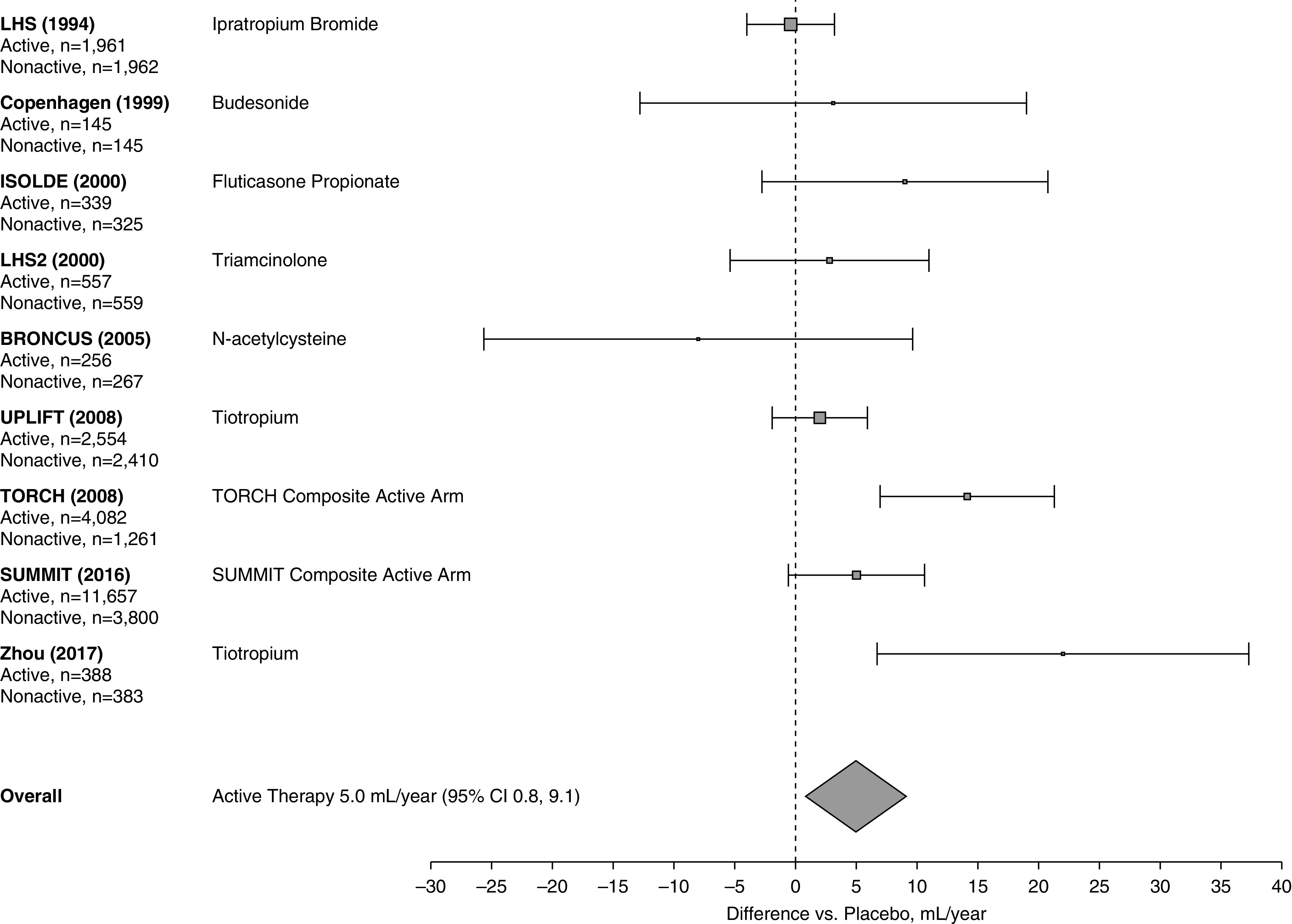
Effect of all active therapies on the rate of decline in FEV_1_. The center of the diamond indicates the point estimate and the width is the 95% CI. BRONCUS = Bronchitis Randomized on NAC Cost-Utility Study; CI = confidence interval; COPD = chronic obstructive pulmonary disease; ISOLDE = Inhaled Steroids in Obstructive Lung Disease in Europe; LHS = Lung Health Study; LHS2 = Lung Health Study 2; SUMMIT = Study to Understand Mortality and Morbidity; TORCH = Toward a Revolution in COPD Health; UPLIFT = Understanding Potential Long-Term Impacts on Function with Tiotropium.

Table 2 shows the values of FEV_1_ decline for the placebo and active arms in each of the individual studies included in this review. As shown in Figure 2, the overall active treatment arms demonstrated a 5.0-ml/yr reduction (95% CI, 0.8–9.1 ml/yr; *P* < 0.001) in the rate of FEV_1_ decline compared with the placebo arms. Figure E1 shows the results of the same analysis, excluding the BRONCUS study because it used the oral agent *N*-acetylcysteine. This analysis demonstrated a larger difference in favor of all active components compared with placebo of 5.6 ml/yr (95% CI, 1.3–9.8).

**Table 2.** Impact of Therapy on FEV_1_, in the Studies Included in This Systematic Review

	(1997–2003) BRONCUS* Ref 16	(1992–1998) ISOLDE* Ref 6	(2000–2005) TORCH* Ref 18	(2003–2008) UPLIFT* Ref 11	(1986–1994) Lung Health I* Ref 3	(2011–2015) Zhou *et al*.* Ref 19	(2011–2015) SUMMIT* Refs 12 and 20	(1992–1994) Copenhagen City Lung Study* Ref 17	(1994–1999) Lung Health Triamcinolone* Ref 21
Treatment arms: placebo**/active intervention**	Placebo**/*N*-acetylcysteine**	Placebo**/fluticasone propionate**	Placebo**/salmeterol/fluticasone propionate/salmeterol + fluticasone propionate/*TORCH composite active arm***	Placebo**/tiotropium**	Placebo/**ipratropium bromide**	Placebo**/tiotropium**	Placebo**/fluticasone furoate/vilanterol/fluticasone furoate + vilanterol/*SUMMIT composite active arm***	Placebo**/budesonide**	Placebo**/triamcinolone acetonide**
FEV_1_ decline in placebo/active (SE), ml	47 (6)/**54 (**6**)**	59 (4.4)/**50 (4.1)**	55.3 (3.2)/**42.3 (3.1)/42.3 (3.1)/39.0 (3.0)/*41.2 (1.8)***	42 (1)/**40 (**1**)**	52.7 (1.3)/**56.2 (1.3)**	51 (6)/**29 (**5**)**	46 (2.5)/**38 (2.4)/47 (2.4)/38 (2.4)/*41 (1.4)***	49.6/**46.0**	47.0 (3.0)/**44.2 (2.9)**
Treatment difference for each active arm vs. placebo (SE) [95% CI], ml	**−8 (9.0) [−25 to 10]**	**9 (6.0) [−3 to 20]**	**13.0 (4.4) [4.3 to 21.7]/13.0 (4.4) [4.3 to 21.7]/16.3 (4.4) [7.7 to 24.9]/*14.1 (3.7) [7.0 to 21.3]***	**2 (**2**) [−2 to 6]**	**−4 (1.8)**	**22 (7.8) [6 to 37]**	**8 (3.5) [1 to 14]/−2 (3.4) [−8 to 5]/8 (3.4) [1 to 15]/*5 (2.9) [−1 to 11]***	**3.1 (8.1) [−12.8 to 19.0]**	**2.8 (4.2)**

*Definition of abbreviations*: BRONCUS = Bronchitis Randomized on NAC Cost-Utility Study; CI = confidence interval; COPD = chronic obstructive pulmonary disease; ISOLDE = Inhaled Steroids in Obstructive Lung Disease in Europe; Ref = reference; SUMMIT = Study to Understand Mortality and Morbidity; TORCH = Toward a Revolution in COPD Health; UPLIFT = Understanding Potential Long-Term Impacts on Function with Tiotropium.

The table presents the rates and the differences between the treatment arms. Statements and numbers in bold reflect the active arms of the study.

* Date is start of recruitment to study complete.

Table E2 shows the effects of therapy on exacerbations and SGRQ for all treatment arms in the studies included in this analysis.

**Figure 3.**
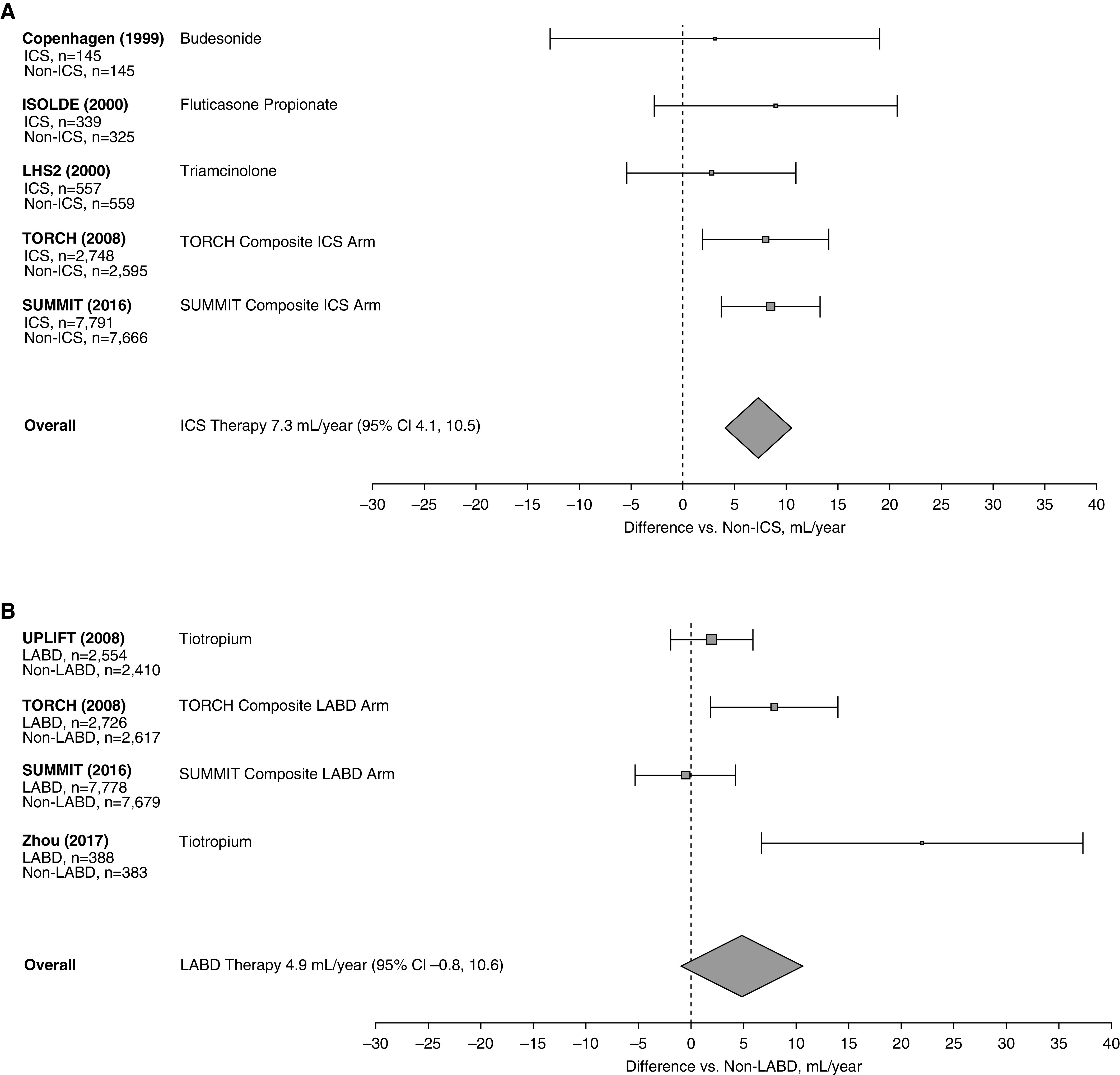
(*A*) Effect of ICS-containing therapies on the rate of decline in FEV_1_. The center of the diamond indicates the point estimate and the width is the 95% confidence interval (CI). (*B*) Effect of inhaled LABD therapy on the rate of decline in FEV_1_. The center of the diamond indicates the point estimate and the width is the 95% CI. COPD = chronic obstructive pulmonary disease; ICS = inhaled corticosteroid; ISOLDE = Inhaled Steroids in Obstructive Lung Disease in Europe; LABD = long-acting bronchodilator; LHS2 = Lung Health Study 2; SUMMIT = Study to Understand Mortality and Morbidity; TORCH = Toward a Revolution in COPD Health; UPLIFT = Understanding Potential Long-Term Impacts on Function with Tiotropium.

Figure 3A shows that the difference between the studies with an active ICS or ICS/bronchodilator-containing arms and placebo was 7.3 ml/yr (95% CI, 4.1 to 10.5), whereas Figure 3B shows that the difference between LABD-containing arms against placebo was 4.9 ml/yr (95% CI, −0.8 to 10.6). Of note, in the UPLIFT trial, 60% of the patients randomized to placebo were already taking long-acting β-agonists.

## Discussion

This systematic review shows that pharmacological therapy modifies disease progression as expressed by the rate of decline in FEV_1_ in patients with COPD. Although the yearly difference may seem small in absolute numbers, its significance over time is important in a disease in which progression is measured in decades.

The most recent Global Initiative for Chronic Obstructive Lung Disease (GOLD) report states that pharmacotherapy is effective in reducing symptoms and exacerbations and improving quality of life and exercise endurance, but there is no conclusive evidence that it modifies the long-term decline in lung function (22), and this view is reflected in the most recent guidance on the pharmacological treatment of COPD published by the American Thoracic Society (23). Based on the observation that some *post hoc* analyses of large studies had suggested an effect of ICS on rate of FEV_1_ decline, the GOLD document states that trials should be specifically designed to test whether ICS can achieve such an effect. However, given the benefit that inhaled medications provide patients with COPD in outcomes such as exacerbations, dyspnea, and health status, it would be unethical to subject symptomatic patients with COPD to trials that include a placebo-controlled arm for studies that may last over several years.

The first consideration worth addressing from the results is the significance of the average difference of 5.0 ml/yr in FEV_1_ between active medications and placebo. A good starting point is to compare this difference with the changes reported in the same studies (where the same issues of differential dropout and patient selection apply) for other patient-related outcomes, such as health status and exacerbations, which have led to the acceptance of pharmacotherapy as being effective in COPD. This comparison is difficult because the units used to measure the different outcomes differ, and their interpretation may be difficult to judge. With all these limitations, it is interesting to note that the differential benefit observed for FEV_1_ is within the range of the benefits reported for health status and for exacerbation rate shown in Table E2. Indeed, although a relative decrease of exacerbation in the same studies ranges from 3% to 43%, and that of the SGRQ ranges between 1 and 3 units in a scale from 0 to 100, the FEV_1_ difference ranges from 4% to 43% of the rate of FEV_1_ decline observed in the placebo arm in those studies. No comparison was made with exercise endurance in that table because this was not reported in any of the studies analyzed. However, several studies evaluating the benefit of pharmacological agents on exercise endurance compared with placebo using a constant load cardiopulmonary exercise test show a proportional increase that ranges from 3% to 10% in favor of the active medication (24, 25).

It is important to note that the FEV_1_ decline benefit observed in the studies included may underestimate the true effect of the medications on many patients because close to half of patients who are diagnosed as having COPD in the sixth decade do so without a rapid decline in lung function and thus are unlikely to normalize an already normal rate of lung function decline (9). Although one of the studies included (26) suggested that blood eosinophil level might influence the beneficial effect of FEV_1_ progression, the lack of this variable in the larger data set precludes any firm conclusion on this potential relationship. As seen in Table 1, in all studies included, the dropout rate was larger in the placebo compared with the active arm. Patients who drop out of pharmacological trials tend to be sicker, with worse outcomes in all variables tested, whereas the remaining patients in the placebo arm are usually healthier, therefore tending to bring all variables to the projected mean change for that variable (27, 28), an effect that may be partially adjusted by the use of the random effects models, as was done in most of those studies. In addition, the mean age of patients recruited into the studies included was approximately 64 years, when most of the course of COPD has been run and modification of clinical outcomes is more difficult to obtain. This is important because the age at which therapy is initiated influences the degree of response. Indeed, the study by Morice and colleagues (29) showed that in 356 patients younger than 50 years in the UPLIFT study, there was a mean decline in post-bronchodilator FEV_1_ that was 58 ml/yr for placebo compared with 38 ml/yr for tiotropium, a 20-ml/yr difference. This suggests that if therapy was initiated earlier (i.e., at 50 yr), the putative 100-ml minimal important difference threshold could be reached in 5 years. It is also likely that response to therapy varies and that specific subgroups may experience even better outcomes. That this seems to be the case is suggested by the larger benefit observed in patients with moderate airflow limitation (GOLD 2) compared with severe and very severe obstruction (GOLD 3 and 4) in those studies in which either by study design or by *post hoc* analysis the response was evaluated in relation to airflow limitation severity (19, 30). Finally, the true difference between the absolute improvement in FEV_1_ between active treatment and placebo is larger because all pharmacological therapies include a bronchodilator effect that averages close to 100 ml for the drugs studied.

Data for individual components could only be completed for ICS, ICS-containing combinations, and LABD because those were the only studies with enough subject numbers to provide meaningful data. As can be seen in Figure 3, the difference was statistically significant for the ICS-containing arms compared with placebo, and although not significant for LABD, it may have reached significance if there were more long-term studies for these drugs and if 60% of the patients in the UPLIFT study using tiotropium had not been on long-acting β-adrenergic agents. The exclusion of the only study using the oral medication *N*-acetylcysteine resulted in an average reduction of 5.9 ml/yr in the rate of FEV_1_ decline compared with the placebo arms (Figure E1).

We cannot address the reasons why the different treatments might influence the rate of decline of FEV_1_, although as we illustrate, treatment reduces exacerbation rates, which are associated with lung function decline (8, 31). Recent data have shown that maximizing the therapy that is usually given to patients with COPD and more severe disease and exacerbations can reduce the risk of these events and of dying (32, 33); these findings are consistent with this complementary evidence from the published literature.

This systematic review is useful because it addressed one single research question, used studies with a large number of patients, had a high-quality control of the primary outcome (FEV_1_), and followed patients over a long enough period of time to provide an answer to the research question. It also addresses a very important need to modify disease progression, for which there will unlikely be a prospective study, given the ethical implications for its implementation. However, there are limitations that are inherent to any review. Some studies cannot be included because they do not meet the selection criteria. One such study, European Respiratory Society Study on Chronic Obstructive Pulmonary Disease (EUROSCOPE), deserves a special comment (5). It specifically addressed the question posed here, comparing the FEV_1_ decline over 3 years between inhaled budesonide and placebo. However, the study could not be included because the results were reported as median values without their variance. However, in that study, the FEV_1_ decline in the active arm was 57 ml/yr compared with 69 ml/yr in placebo-treated patients, in agreement with our overall results. Finally, reviews cannot provide adequate comparisons of the treatment effects of the different medications. However, the CI in the larger studies provides a good approximation to the average effect of each of the components included in the analysis.

### Conclusions

Our analyses show that pharmacotherapy is effective in altering rate of lung function decline and that the yearly absolute difference observed is similar to the treatment difference reported for clinical outcome such as health status and exacerbations. Current guidelines should be adjusted to reflect these findings, and future studies should be directed to evaluate the potential benefits in patients likely to benefit, such as those with rapid lung function decline.

## Supplementary Material

Supplements

Author disclosures
