# Supplementary material for: Pharmacotherapy and Lung Function Decline in Patients with Chronic Obstructive Pulmonary Disease. A Systematic Review
Source: Am J Respir Crit Care Med. 2021 Mar 15;203(6):689–98. doi: 10.1164/rccm.202005-1854OC (PMC7958521; doi:10.1164/rccm.202005-1854OC)
Supplement: Supplements [file rccm.202005-1854OC.html]

Pharmacotherapy and Lung Function Decline in Patients with Chronic Obstructive Pulmonary Disease. A Systematic Review | American Journal of Respiratory and Critical Care Medicine

- celli\_data\_supplemement.pdf (821 KB)
- disclosures.pdf (280 KB)
