# Supplementary material for: Pharmacotherapy and Lung Function Decline in Patients with Chronic Obstructive Pulmonary Disease. A Systematic Review
Source: Am J Respir Crit Care Med. 2021 Mar 15;203(6):689–98. doi: 10.1164/rccm.202005-1854OC (PMC7958521; doi:10.1164/rccm.202005-1854OC)
Supplement: Supplements [file rccm.202005-1854OC_celli_data_supplemement.pdf]

Pharmacotherapy and Lung Function Decline in Patients with Chronic Obstructive Pulmonary Disease: A Systematic Review

Bartolome R. Celli, Julie A. Anderson, Nicholas J. Cowans, Courtney Crim, Benjamin F. Hartley, Fernando J. Martinez, Andrea N. Morris, Holly Quasny, Julie Yates, Jørgen Vestbo, and Peter M.A. Calverley

ONLINE DATA SUPPLEMENT

Figure E1. Effect of active therapy on the rate of decline in FEV_1. Sensitivity analysis with the BRONCUS study using N-acetyl-cysteine removed. The center of the diamond indicates the point estimate and the width is the 95% confidence interval.

TABLE E1. PubMed search strategy

**Search terms**	
**#1 COPD**	“Pulmonary Disease, Chronic Obstructive"[Mesh] OR chronic obstructive pulmonary disease*[tiab] OR chronic obstructive airway disease*[tiab] OR chronic obstructive lung disease*[tiab] OR chronic bronchitis[tiab] OR emphysema[tiab] OR chronic airflow obstruction[tiab] OR COPD[tiab] OR COAD[tiab]
**#2 Decline in lung function/FEV1**	(("Forced Expiratory Volume"[Mesh] OR Forced Expiratory Volume*[tiab] OR FEV1[tiab] OR FEV_1[tiab] OR Timed Vital Capacit*[tiab] OR lung function*[tiab] OR lungfunction*[tiab] OR respiratory function*[tiab] OR pulmonary function*[tiab]) AND (decline*[tiab] OR decrease*[tiab] OR diminish*[tiab] OR reduc*[tiab] OR low[tiab] OR lower[tiab] OR worsen*[tiab] OR change*[tiab] OR development*[tiab] OR trajector*[tiab] OR "Disease Progression"[Mesh] OR progress*[tiab] OR deteriorat*[tiab] OR natural history[tiab]))
**#3 Study designs (i.e. meta-analysis)**	meta-analysis[pt] OR meta-analysis[tiab] OR meta-analyses[tiab] OR meta analysis[tiab] OR meta analyses[tiab] OR metaanalysis[tiab] OR metaanalyses[tiab] OR randomized controlled trial[pt] OR controlled clinical trial[pt] OR randomized[tiab] OR randomised[tiab] OR RCT[tiab] OR controlled[tiab] OR placebo*[tiab] OR trial[tiab] OR intervention[tiab] OR "Cross-Over Studies"[Mesh] OR "Double-Blind Method"[Mesh] OR "Prospective Studies"[Mesh] OR “Follow-up Studies”[Mesh] OR “Cohort Studies”[Mesh] OR crossover[tiab] OR cross-over[tiab] OR double-blind[tiab] OR doubleblind[tiab] OR single-blind[tiab] OR singleblind[tiab] OR cohort*[tiab] OR prospective[tiab] OR longitudinal[tiab] OR observational[tiab] OR follow-up[tiab] OR followup[tiab]
**Limits**	No country limits<br>No time limits<br>Language: English

TABLE E2. Impact of Therapy on St. George's Respiratory Questionnaire Scores and Exacerbations Rate in the Studies Included in this Systematic Review. The Table Presents the Rates and the Differences Between the Treatment Arms

	(1997-2003) BRONCUS* Ref # 16	(1992-1998) ISOLDE* Ref # 6	(2000-2005) TORCH* Ref # 10	(2003-2008) UPLIFT* Ref # 11	(1996-1994) Lung Health I* Ref # 3	(2011-2015) Zhou et al* Ref # 19	(2011-2015) SUMMIT* Refs # 12, 20	(1992-1994) Copenhagen City Lung Study* Ref # 17	(1994-1999) Lung Health Triamcinolone* Ref # 21
Treatment arms: placebo or usual care / **active intervention(s)**	Placebo / **N-acetylcysteine**	Placebo / **fluticasone propionate**	Placebo / **salmeterol / fluticasone propionate / salmeterol + fluticasone propionate**	Placebo / **tiotropium**	Placebo / **ipratropium bromide**	Placebo / **tiotropium**	Placebo / **fluticasone furoate / vilanterol / fluticasone furoate + vilanterol**	Placebo / **budesonide**	Placebo / **triamcinolone acetonide**
SGRQ – score change at 1 year in placebo / **active** (SE)	1.24 (0.49) / **1.45 (0.47)**	3.17 (0.31) / **2.00 (0.29)**	0.2 (0.37) / **-0.8 (0.35) / -1.8 (0.35) / -3.0 (0.35)** †	1.21 (0.09) / **1.25 (0.09)**	NA	NA	NA	NA	NA
Treatment difference in score for each active arm vs placebo (SE) [95% CI]	0.21 [-1.13–1.55]	−1.17 (0.40) [−1.95 to −0.39]	-1.0 (0.15) [-2.0–0.0] / -2.0 (0.49) [-2.9 to -1.0] / -3.1 (0.51) [-4.1 to -2.1]	0.04 (0.13) [-0.2–0.3]	NA	NA	NA	NA	NA
Exacerbations rate per year in placebo / **active** (SE)	1.31 / **1.25**	1.32 [0–30] / **0.99 [0–26]**	1.13 / **0.97 / 0.93 / 0.85**	0.85 (0.02) / **0.73 (0.02)**	NA	0.50 (0.05) / **0.27 (0.03)**	0.35 / **0.31 / 0.31 / 0.25**	161 / **155**‡	NA
Treatment ratio for each active arm vs. placebo	0.990 [0.889–1.101]	−0.3 [−0.4–0.0] §	0.85 [0.78–0.93] / 0.82 [0.76–0.89] /	0.86 [0.81–0.91]	NA	0.53 [0.39–0.73]	12% [4%–19%] / 10% [2%–18%] /	NA	NA

(SE) [95% CI]			0.75 [0.69–0.81]				29% [22%–35%] ll		

BRONCUS = Bronchitis Randomized on NAC Cost-Utility Study; CI = confidence interval; ISOLDE = Inhaled steroids in obstructive lung disease in Europe; SE, standard error; SGRQ = St George's Respiratory Questionnaire; SUMMIT = Study to Understand Mortality and Morbidity TORCH = Towards a Revolution in COPD Health; UPLIFT = Understanding Potential Long-Term Impacts on Function with Tiotropium.

* Date is start of recruitment to study complete.
† Overall (not at 1 year).
‡ Number in study per arm difference not significant.
§ Treatment difference not ratio.
ll Rate reduction shown.

Figure E1

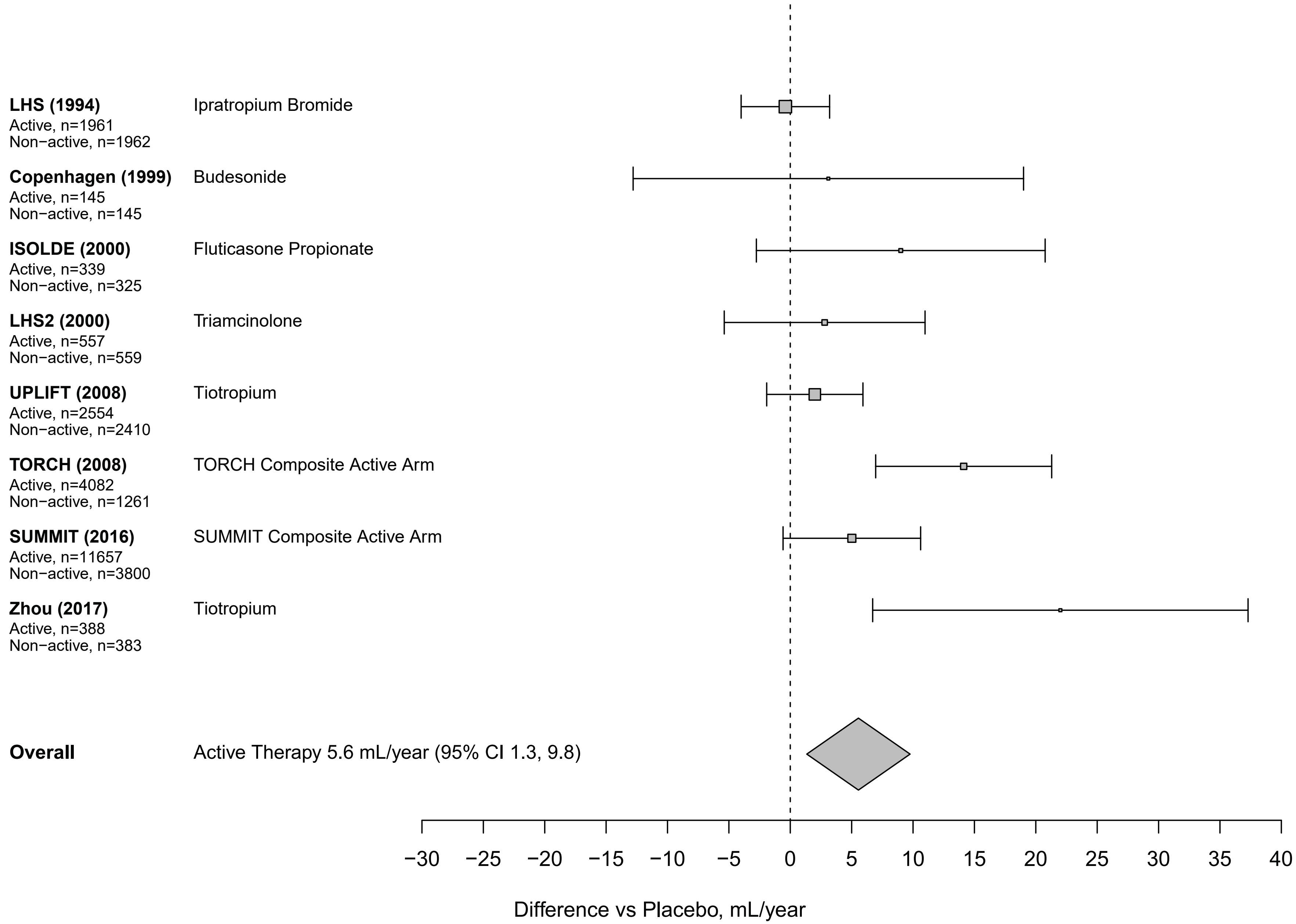

LHS (1994)
Active, n=1961
Non−active, n=1962
Ipratropium Bromide
Copenhagen (1999)
Active, n=145
Non−active, n=145
Budesonide
ISOLDE (2000)
Active, n=339
Non−active, n=325
Fluticasone Propionate
LHS2 (2000)
Active, n=557
Non−active, n=559
Triamcinolone
UPLIFT (2008)
Active, n=2554
Non−active, n=2410
Tiotropium
TORCH (2008)
Active, n=4082
Non−active, n=1261
TORCH Composite Active Arm
SUMMIT (2016)
Active, n=11657
Non−active, n=3800
SUMMIT Composite Active Arm
Zhou (2017)
Active, n=388
Non−active, n=383
Tiotropium
Overall
Active Therapy 5.6 mL/year (95% CI 1.3, 9.8)
−30 −25 −20 −15 −10 −5 0 5 10 15 20 25 30 35 40
Difference vs Placebo, mL/year
